# APOE Genotype Modifies the Plasma Oxylipin Response to Omega-3 Polyunsaturated Fatty Acid Supplementation in Healthy Individuals

**DOI:** 10.3389/fnut.2021.723813

**Published:** 2021-09-17

**Authors:** Rasha N. M. Saleh, Annette L. West, Annika I. Ostermann, Nils Helge Schebb, Philip C. Calder, Anne Marie Minihane

**Affiliations:** ^1^Nutrition and Preventive Medicine Group, Norwich Medical School, University of East Anglia, Norwich, United Kingdom; ^2^Department of Clinical and Chemical Pathology, Faculty of Medicine, Alexandria University, Alexandria, Egypt; ^3^School of Human Development and Health, Faculty of Medicine, University of Southampton, Southampton, United Kingdom; ^4^Chair of Food Chemistry, Faculty of Mathematics and Natural Sciences, University of Wuppertal, Wuppertal, Germany; ^5^National Institute for Health Research (NIHR) Southampton Biomedical Research Centre, University Hospital Southampton NHS Foundation Trust and University of Southampton, Southampton, United Kingdom

**Keywords:** *APOE*, oxylipins, PUFAs, EPA, DHA, HDHA, HEPE, PPAR

## Abstract

The omega-3 polyunsaturated fatty acids (n-3 PUFAs), eicosapentaenoic acid (EPA) and docosahexaenoic acid (DHA), mediate inflammation in large part by affecting pro-inflammatory and anti-inflammatory/pro-resolving oxylipin concentrations. Common gene variants are thought to underlie the large inter-individual variation in oxylipin levels in response to n-3 PUFA supplementation, which in turn is likely to contribute to the overall heterogeneity in response to n-3 PUFA intervention. Given its known role in inflammation and as a modulator of the physiological response to EPA and DHA, here we explore, for the first time, the differential response of plasma hydroxy-, epoxy- and dihydroxy-arachidonic acid, EPA and DHA oxylipins according to apolipoprotein E (*APOE*) genotype using samples from a dose-response parallel design RCT. Healthy participants were given doses of EPA+DHA equivalent to intakes of 1, 2, and 4 portions of oily fish per week for 12 months. There was no difference in the plasma levels of EPA, DHA or ARA between the wildtype *APOE3/E3* and *APOE4* carrier groups after 3 or 12 months of n-3 PUFA supplementation. At 12 months, hydroxy EPAs (HEPEs) and hydroxy-DHAs (HDHAs) were higher in *APOE4* carriers, with the difference most evident at the highest EPA+DHA intake. A significant APOE^*^n-3 PUFA dose effect was observed for the CYP-ω hydroxylase products 19-HEPE (*p* = 0.027) and 20-HEPE (*p* = 0.011). 8-HEPE, which, along with several other plasma oxylipins, is an activator of peroxisome proliferator activated receptors (PPARs), showed the highest fold change in *APOE4* carriers (14-fold) compared to *APOE3/E3* (4-fold) (*p* = 0.014). Low basal plasma EPA levels (EPA < 0.85% of total fatty acids) were associated with a greater change in 5-HEPE, 9-HEPE, 11-HEPE, and 20-HEPE compared to high basal EPA levels (EPA > 1.22% of total fatty acids). In conclusion, *APOE* genotype modulated the plasma oxylipin response to increased EPA+DHA intake, with *APOE4* carriers presenting with the greatest increases following high dose n-3 PUFA supplementation for 12 months.

## Introduction

**Figure 1:**
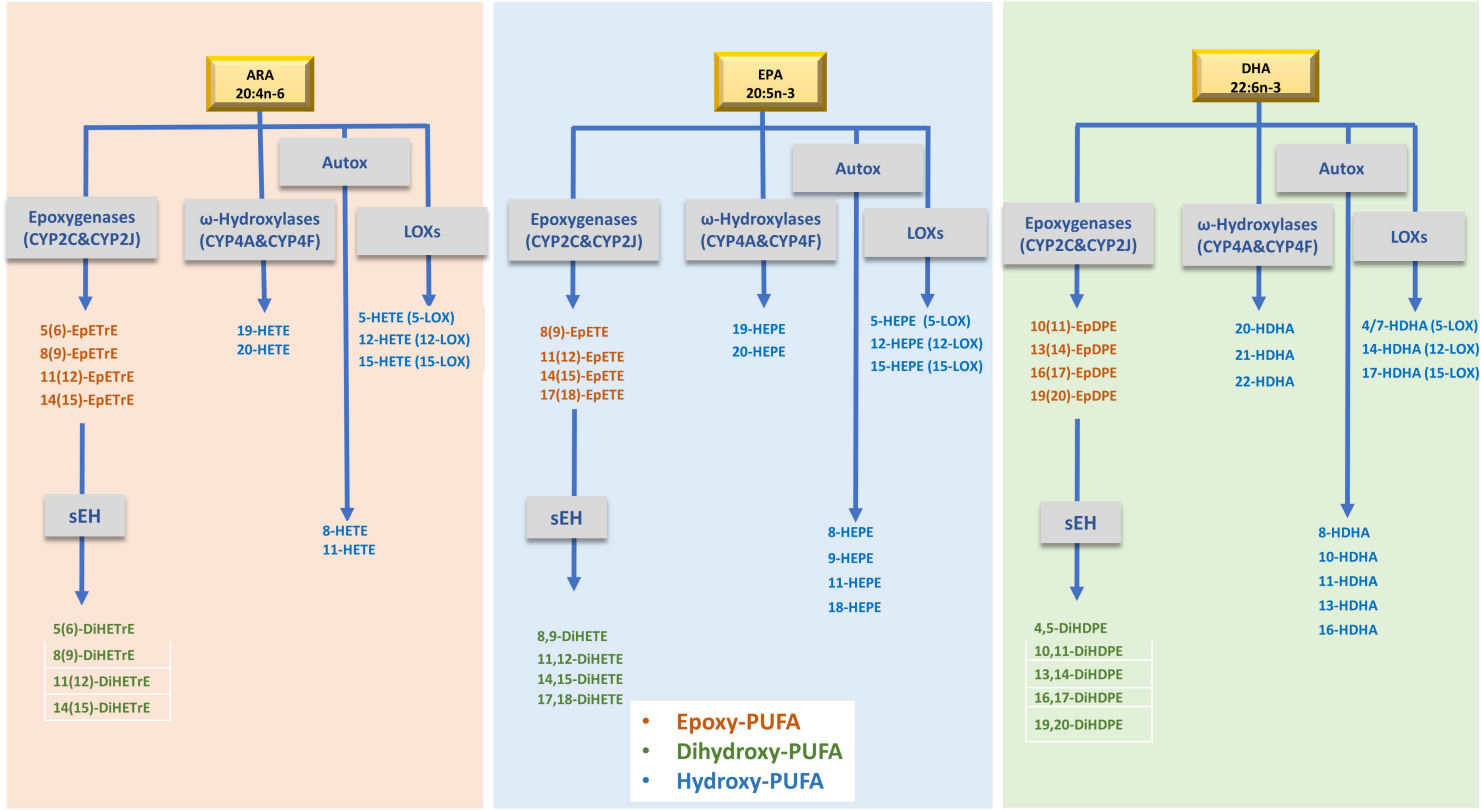
Simplified metabolism of ARA, EPA and DHA by, LOXs and CYPs to produce the oxylipins evaluated in this study. ARA, Arachidonic acid; EPA, Eicosapentaenoic acid; DHA, docosahexaenoic acid; LOXs, lipoxygenases; sEH, soluble epoxide hydrolase enzyme; EpETrE, epoxyeicosatrienoic acid; DiHETrE, dihydroxyeicosatrienoic acid; HETE, hydroxy-eicosatetraenoic acid; EpETE, epoxyeicosatetraenoic acid; DiHETE, dihydroxyeicosatetraenoic acids; HEPE, hydroxyeicosapentaenoic acid; EpDPE, epoxydocosapentaenoic acid; DiHDPE, dihydroxydocosapentaenoic acid; HDHA, hydroxydocosahexaenoic acid. Of note, several oxylipins can be formed by different routes as well as by chemical autoxidation.

The omega-3 polyunsaturated fatty acids (n-3 PUFAs) eicosapentaenoic acid (EPA) and docosahexaenoic acid (DHA) have long been known to play a role in promoting human health and well-being (1). Higher EPA and DHA intake is associated with a lower risk of cardiovascular disease and mortality (2, 3), cognitive decline (4), rheumatoid arthritis (5), obesity (6), and overall mortality (7). The biological actions of n-3 PUFAs are partly mediated through their oxidized metabolites, called oxylipins. Oxylipins are formed via three main pathways involving cyclooxygenases, lipoxygenases, and several cytochrome P450 (CYP) enzymes which produce hydroxy-, dihydroxy-, or epoxy- fatty acids (FAs) among other products. Due to their highly unsaturated status, PUFAs can also be non-enzymatically oxidized (i.e., autooxidation) by reactive oxygen and nitrogen species, to produce a number of oxylipins (Figure 1) (8, 9). Oxylipins are potent lipid mediators of multiple physiological processes (10). Epoxy-arachidonic acid (ARA) species (EpETrEs), products of CYP2C and 2J epoxygenases, have recently been shown to have cardiovascular (11, 12) and anti-inflammatory benefits (13). Epoxy-EPAs (EpETEs) and -DHAs (EpDPEs) are anti-arrhythmic (14) and inhibit angiogenesis (15). Epoxy-FAs are metabolized by hydration to the corresponding less active dihydroxy-FAs by the action of soluble epoxide hydrolase (sEH) (9). As a result, the ratio of dihydroxy- to epoxy-FAs has been used as an indicator of sEH activity (16). Hydroxy-ARAs (HETEs), -EPAs (HEPEs), and -DHAs (HDHAs) have a wide range of functions, for example regulating neutrophil chemotaxis, platelet aggregation and adipogenesis (9). 8-HEPE has recently been found to reduce plasma LDL-cholesterol and triglycerides in obese mice through binding to peroxisome proliferation activator receptors (PPARs) (17). Other hydroxy-FAs, such as 18-HEPE and 17-HDHA are precursors for specialized pro-resolving mediators (SPMs). SPMs (including resolvins, protectins, maresins, and lipoxins) are now known for their anti-inflammatory and pro-resolving roles (18).

Several intervention studies have shown a rise in EPA- and DHA-derived and a fall in ARA-derived oxylipins in response to n-3 PUFA supplementation (19–23). This response is linearly related to n-3 PUFA dose (24). However, despite the high compliance to n-3 PUFA treatment in most studies, a strong inter-individual variation in the oxylipin response to different doses of n-3 PUFA intervention was observed (23, 25, 26). This variation has been partly explained by differences in baseline EPA and DHA status. Individuals with lower basal levels of EPA and DHA levels showed a greater increase in the n-3 PUFA-derived oxylipins in response to increased n-3 PUFA intake (23, 25). Genetic variation in enzymes involved in PUFA metabolism have been implicated as another possible cause of variation in the oxylipin response to n-3 PUFAs. Genetic variation in LTA_4_H, an enzyme in the pathway of leukotriene synthesis, significantly interacted with dietary intake of n-3 and n-6 fatty acids to determine intima-media thickness (IMT) in one population (27). In another study, variants in *ALOX5* gene were associated with a differential oxylipin response to fish oil supplementation (28).

Apolipoprotein E (APOE) regulates the concentrations and metabolism of cholesterol and PUFAs in the circulation and in tissues (29). The *APOE* gene has three allele variants ε2, ε3, and ε4, determined by two SNPs, rs429358 at codon 112 and rs7412 at codon 158. The frequency of the major *APOE3* allele ranges from 48 to 94% while the *APOE4* allele has a wider global range (3–41%) (30). *APOE* genotype has long been known to affect the response to n-3 PUFA interventions in healthy participants (31) and in patients with cardiovascular (32) and cognitive disorders (33). Studies have investigated the benefit of *APOE4*-targeted dietary approaches on blood lipid levels (34, 35) and Alzheimer's disease risk (36, 37). Despite *APOE* genotype being a known modulator of response to n-3 PUFA interventions, the mechanistic basis for this is poorly understood, and the effect of *APOE* genotype on oxylipin responses to increased n-3 PUFA intake has not been investigated. In this study, we hypothesize that the change in the plasma concentrations of free oxylipins in response to n-3 PUFA supplementation will differ according to *APOE* genotype. To test this hypothesis, we genotyped healthy subjects who participated in a well-designed randomized control trial (RCT), where EPA and DHA capsules were given in different doses to mimic three different patterns of oily fish intake for a duration of 12 months. Plasma phosphatidylcholine (PC) fatty acids and free oxylipin concentrations were measured at baseline, 3 and 12 months, as reported elsewhere (24, 38).

## Methods

The primary aim of the RCT was to investigate the time course and dose-response effect of EPA and DHA supplementation on the EPA and DHA content of different blood and tissue pools (38); a secondary a posteriori aim was to determine the effect on plasma oxylipin concentrations (24). Here we investigate if *APOE* genotype influences habitual plasma oxylipin concentrations and their response to EPA and DHA supplementation. The current analysis is considered as exploratory.

### Participants and Study Design

The study was a double-blinded, parallel RCT in healthy subjects with low habitual fish intake. The study protocol and all procedures and analyses were approved by the Suffolk Local Research Ethics Committee (approval 05/Q0102/181) with the participant consent process allowing for additional analysis of the data collected or biobanked samples. The trial is registered at www.controlled-trials.com as ISRCTN48398526. The study design and the characteristics of the study participants have been described elsewhere (38). Briefly, 163 participants were given EPA+DHA (as triglycerides) in capsules with weekly doses equivalent to the consumption of 0, 1, 2, or 4 portions of fatty fish per week, with one portion being equivalent to 3.27 g EPA + DHA (1:1.2, wt:wt). The period of supplementation was 12 months and blood was sampled at baseline, 3 months and 12 months. Buffy coat and plasma were prepared. For the current analysis, a subset of 110 subjects with *APOE* genotype data were selected according to the availability of a buffy coat for DNA extraction and *APOE* genotyping. The characteristics of the study population based on their *APOE* genotype are presented in Table 1.

**Table 1:** Basic characteristics of the study population at baseline based on *APOE* genotype.

	***APOE2* (*n* = 18)**	***APOE3* (*n* = 66)**	***APOE4* (*n* = 26)**
Age (years)	55.2± 15.1^a^	49.6 ± 15.9^a^	48.5 ± 13.8^a^
Gender (M/F)	6/12	35/31	12/14
BMI (kg/m^2^)	24.9 ± 3.7^a^	25.7 ± 3.8^a^	25.3 ± 4.3^a^

a *Mean ± SD*.

### DNA Extraction and *APOE* Genotyping

DNA was extracted from the buffy coat of whole blood using the QiAmp DNA Blood Mini kit (Qiagen, UK). The quality and quantity of extracted DNA was checked using a Nanodrop 2000 spectrophotometer. Samples with a yield of at least 15 ng/μl and a 260/280 ratio of at least 1.8 were used for subsequent genotyping.

*APOE* genotyping was performed by LGC Genomics Ltd, Hoddesdon, UK, using the KASP technology. Primers were designed for the two single-nucleotide polymorphisms (SNPs) in the *APOE* gene; rs429358 at codon 112 and rs7412 at codon 158. These two SNPs determine the *APOE2, E3*, and *E4* alleles.

### Oxylipin Analysis

Plasma free oxylipins were measured at baseline, 3 and 12 months of n-3 PUFA supplementation. Oxylipin analysis was performed as described elsewhere (24, 39). Briefly, oxylipins were isolated from plasma using Bond Elut Certify II Cartridges (Agilent) and analyzed by liquid chromatography-tandem mass spectrometry (LC-MS/MS) after negative electrospray ionization in scheduled selected reaction monitoring.

Fifty EPA-, DHA-, and ARA-derived oxylipins were included in the present study: 9 hydroxy-EPAs (HEPEs), 4 dihydroxy-EPAs (DiHETEs), 3 epoxy-EPAs (EpETEs), 11 hydroxy-DHAs (HDHAs), 5 dihydroxy-DHAs (DiHDPEs), 4 epoxy-DHAs (EpDPEs), 6 hydroxy-ARAs (HETEs), 4 dihydroxy-ARAs (DiHETrEs), and 4 epoxy-ARAs (EpETrEs). The concentrations of all HEPEs, DiHETEs, EpETEs, HDHAs, DiHDPEs, EpDPEs, HETEs, DiHETrEs, and EpETrEs covered by the analytical method were summed from the individual data as described previously (24).

### Fatty Acids Analysis

ARA, EPA, and DHA were measured in the plasma phosphatidylcholine (PC) fraction as described previously (38). Briefly, total lipid was extracted from plasma using chloroform:methanol (2:1) Plasma lipid fractions were separated and isolated by solid-phase extraction on aminopropylsilica cartridges. PC, which is the major plasma phospholipid, was eluted with chloroform:methanol (60:40, vol:vol). Fatty acid methyl esters (FAMEs) were formed by transesterification with methanol in sulphuric acid and were separated using gas chromatography. FAMEs were identified by comparison with authentic standards. Fatty acids are expressed as weight percent of total fatty acids in plasma PC.

### Statistics and Data Analysis

Data for fatty acids, oxylipins, and *APOE* genotyping were processed using RStudio. Results are presented as mean ± SEM. The absolute changes in oxylipin concentration after 12 months of supplementation were calculated as conc(t12)-conc(t0). Relative changes after 12 months were calculated as conc(t12)/conc(t0). Percent relative change of EPA and DHA and their derived oxylipins were calculated as conc(t12)/ conc(t0)^*^100.

Variables were checked for normality using the Shapiro-Wilk test. For normally distributed variables, an independent sample *t*-test was used to test for significance between *APOE3* (E3/E3) and *APOE4* (E3/E4 + E4/E4) groups. For not-normally distributed variables, the Mann-Whitney test was used. Being aware of the unequal sample sizes between *APOE* groups, Levene's test for homogeneity of variances was conducted. Log transformation was performed when required.

A univariate general linear model was used to investigate the main and combined (interaction) effect of *APOE* genotype and n-3 PUFA dose on the absolute change of individual oxylipin concentrations at 12 months. Age, sex, BMI, and baseline parent n-3 PUFA concentration were used as covariates.

To investigate the effect of *APOE* genotype on the change in oxylipin concentration over time, a repeated measure analysis of oxylipins was performed using the baseline, 3 and 12 month data. A model was built to identify the independent effect of *APOE* genotype and dose as main effects, and “APOE^*^dose” interaction effect. Age, sex and BMI were used as covariates in the model. Time was used as the “within subject” factor. Due to the exploratory nature of this study, correction for multiple testing was not performed and no formal power calculation was carried out, although a retrospective power calculation indicates that for the sum of EPA-, DHA-, and ARA- oxylipins we had 93.6, 91.2, and 53.8% power, respectively, to detect a 5% difference between genotype groups when on the highest dose of n-3 supplementation (4 portions).

All the statistics were carried out using SPSS version 24 (IBM).

## Results

### *APOE* Genotyping Frequencies in the Studied Population

rs429358 and rs7412 were genotyped from the DNA of 110 participants. Basic characteristics of the study population at baseline based on APOE genotype are shown in Table 1. Genotype and allele frequencies, as shown in Table 2, correspond to the frequencies in the European population (39). Using PLINK software (https://www.cog-genomics.org/plink2), all three APOE genotypes were found to be in Hardy-Weinberg equilibrium. Due to the small number of samples with the E2 allele, with numbers of <3 per n-3 PUFA dose group, E2 was excluded from the analysis. Results for E3 (E3/E3 genotype) and E4 (E3/E4 and E4/E4) alleles are thus calculated and displayed hereafter.

**Table 2:** APOE genotype and allele frequencies in the study population.

***APOE* genotype**	**Number**	**Genotype Frequency (%)**	**Allele frequency (%)**
*E2/E2*	2	1.8	E2 = 16.4
*E2/E3*	15	13.6	
*E2/E4*	1	0.9	
*E3/E3*	66	60.0	E3 = 60.0
*E3/E4*	25	22.7	E4 = 23.6
*E4/E4*	1	0.9	
Total	110	100.0	100.0

### Baseline Plasma Levels of ARA, EPA, and DHA, and Their Derived Oxylipins

At baseline, there was no significant effect of *APOE* genotype on plasma PC levels of ARA, EPA, or DHA, or their derived oxylipins except for 11-HDHA and 20-HETE, which were lower in the *APOE4* group (*p* = 0.035 and *p* = 0.04, respectively) (Table 3).

**Table 3:** Baseline plasma phosphatidylcholine fatty acids (% of total fatty acids; %tFA) and oxylipin concentrations (nM) in *APOE3* and *APOE4* individuals.

**Fatty acids and oxylipins**		***APOE3* (*n* = 66)**	***APOE4* (*n* = 26)**	**Total (*n* = 92)**	**P*_**APOE**_***	**P*_**parent PUFA**_***	P_APOE*parent PUFA_
EPA (%tFA)		1.11 ± 0.06	1.19 ± 0.10	1.10 ± 0.05	0.22		
Hydroxy-EPA	5-HEPE	0.36 ± 0.02	0.34 ± 0.03	0.35 ± 0.02	0.84	0.02^*^	0.62
	8-HEPE	0.08 ± 0.00	0.08 ± 0.01	0.08 ± 0.00	0.59	0.01^*^	0.66
	9-HEPE	0.23 ± 0.01	0.23 ± 0.02	0.23 ± 0.01	0.67	0.01^*^	0.72
	11-HEPE	0.11 ± 0.01	0.10 ± 0.01	0.11 ± 0.01	0.36	0.17	0.26
	12-HEPE	0.30 ± 0.03	0.30 ± 0.04	0.30 ± 0.02	0.28	0.58	0.21
	15-HEPE	0.14 ± 0.01	0.15 ± 0.02	0.14 ± 0.00	0.83	0.96	0.97
	18-HEPE	0.19 ± 0.01	0.21 ± 0.02	0.19 ± 0.01	0.51	0.06	0.78
	19-HEPE	1.02 ± 0.06	0.98 ± 0.10	1.00 ± 0.05	0.87	0.10	0.71
	20-HEPE	0.61 ± 0.04	0.68 ± 0.08	0.62 ± 0.03	0.39	0.33	0.62
	sum	3.01 ± 0.16	3.01 ± 0.25	3.01 ± 0.20	0.57	0.11	0.53
Dihydroxy-EPA	8,9-DiHETE	0.09 ± 0.01	0.08 ± 0.01	0.09 ± 0.00	0.93	0.04^*^	0.77
	11,12-DiHETE	0.06 ± 0.00	0.05 ± 0.00	0.06 ± 0.00	0.66	0.02^*^	0.91
	14,15-DiHETE	0.10 ± 0.00	0.09 ± 0.01	0.10 ± 0.01	0.77	0.05	0.57
	17,18-DiHETE	0.66 ± 0.03	0.62 ± 0.04	0.67 ± 0.03	0.61	0.08	0.38
	sum	0.90 ± 0.05	0.85 ± 0.05	0.87 ± 0.05	0.81	0.03^*^	0.54
Epoxy-EPA	11 (12)-EpETE	<0.05	<0.05	<0.05			
	14 (15)-EpETE	0.07 ± 0.01	0.07 ± 0.01	0.07 ± 0.01	0.86	0.12	0.77
	17 (18)-EpETE	<0.1	<0.1	<0.1			
DHA (%tFA) Hydroxy-DHA	3.67 ± 0.12	3.73 ± 0.25	3.69 ± 0.11	0.86			
	4-HDHA	0.45 ± 0.03	0.41 ± 0.03	0.50 ± 0.05	0.41	0.95	0.55
	8-HDHA	0.51 ± 0.03	0.48 ± 0.05	0.50 ± 0.02	0.19	0.22	0.22
	10-HDHA	0.12 ± 0.01	0.12 ± 0.01	0.12 ± 0.00	0.12	0.30	0.12
	11-HDHA	0.23 ± 0.01	0.20 ± 0.02	0.22 ± 0.01	0.04^*^	0.96	0.08
	13-HDHA	0.11 ± 0.01	0.10 ± 0.01	0.10 ± 0.00	0.28	0.17	0.36
	14-HDHA	1.30 ± 0.12	1.08 ± 0.16	1.29 ± 0.11	0.28	0.16	0.40
	16-HDHA	0.17 ± 0.01	0.16 ± 0.01	0.17 ± 0.01	0.57	0.28	0.64
	17-HDHA	0.94 ± 0.05	0.99 ± 0.12	0.98 ± 0.05	0.30	0.51	0.20
	20-HDHA	0.44 ± 0.02	0.43 ± 0.03	0.43 ± 0.02	0.42	0.29	0.40
	21-HDHA	3.28 ± 0.16	3.12 ± 0.30	3.23 ± 0.14	0.35	0.09	0.39
	22-HDHA	2.72 ± 0.16	2.54 ± 0.22	2.75 ± 0.14	0.43	0.66	0.32
	sum	10.43 ± 0.47	10.32 ± 0.82	10.38 ± 0.6	0.16	0.18	0.16
Dihydroxy-DHA	4,5-DiHDPE	1.42 ± 0.07	1.24 ± 0.11	1.37 ± 0.06	0.42	0.99	0.65
	10,11-DiHDPE	0.23 ± 0.01	0.21 ± 0.02	0.23 ± 0.01	0.32	0.33	0.44
	13,14-DiHDPE	0.25 ± 0.01	0.23 ± 0.01	0.26 ± 0.02	0.38	0.14	0.63
	16,17-DiHDPE	0.32 ± 0.01	0.29 ± 0.02	0.33 ± 0.02	0.44	0.11	0.67
	19,20-DiHDPE	3.16 ± 0.13	2.76 ± 0.17	3.18 ± 0.15	0.31	0.05	0.62
	sum	5.48 ± 0.22	5.41 ± 0.57	5.39 ± 0.35	0.43	0.19	0.75
Epoxy-DHA	10 (11)-EpDPE	0.31 ± 0.02	0.28 ±0.03	0.3 ± 0.01	0.25	0.09	0.31
	13 (14)-EpDPE	0.25 ± 0.01	0.24 ± 0.03	0.25 ± 0.01	0.34	0.03^*^	0.36
	16 (17)-EpDPE	0.27 ± 0.01	0.26 ± 0.02	0.26 ± 0.01	0.77	0.05	0.87
	19 (20)-EpDPE	0.48 ± 0.02	0.45 ± 0.04	0.47 ± 0.02	0.25	0.02^*^	0.31
	sum	1.30 ± 0.06	1.30 ± 0.13	1.3 ± 0.10	0.33	0.03^*^	0.39
ARA (%tFA)	9.06 ± 0.23	9.5 ± 0.40	9.19 ± 0.2	0.34			
Hydroxy-ARA	5-HETE	1.35 ± 0.07	1.30 ± 0.09	1.41 ± 0.09	0.91	0.57	0.98
	8-HETE	0.31 ± 0.01	0.31 ± 0.02	0.31 ± 0.01	0.5	0.16	0.41
	11-HETE	0.27 ± 0.01	0.27 ± 0.02	0.27 ± 0.01	0.53	0.41	0.52
	12-HETE	2.05 ± 0.17	2.26 ± 0.37	2.55 ± 0.29	0.52	0.72	0.59
	15-HETE	1.00 ± 0.04	1.03 ± 0.06	1.01 ± 0.03	0.9	0.8	0.85
	20-HETE	0.93 ± 0.05	0.92 ± 0.07	0.93 ± 0.04	0.04^*^	0.74	0.04^*^
	sum	6.21 ± 0.12	6.55 ± 0.09	6.39 ± 0.10	0.41	0.88	0.46
Dihydroxy-ARA	5,6-DiHETrE	0.37 ± 0.04	0.27 ± 0.02	0.34 ± 0.03	0.51	0.82	0.31
	8,9-DiHETrE	0.25 ± 0.01	0.21 ± 0.01	0.24 ± 0.01	0.42	0.20	0.22
	11,12-DiHETrE	0.57 ± 0.02	0.61 ± 0.07	0.58 ± 0.02	0.82	0.19	0.95
	14,15-DiHETrE	0.65 ± 0.02	0.70 ± 0.07	0.66 ± 0.02	0.62	0.19	0.76
	sum	1.84 ± 0.05	1.68 ± 0.06	1.78 ± 0.06	0.46	0.36	0.32
Epoxy-ARA	5 (6)-EpETrE	0.9 ± 0.05	0.8 ± 0.09	0.87 ± 0.04	0.94	0.49	0.90
	8 (9)-EpETrE	0.19 ± 0.01	0.18 ± 0.02	0.19 ± 0.01	0.89	0.19	0.77
	11 (12)-EpETrE	0.20 ± 0.01	0.19 ± 0.01	0.20 ± 0.01	0.56	0.29	0.47
	14 (15)-EpETrE	0.46 ± 0.02	0.42 ± 0.03	0.45 ± 0.02	0.63	0.29	0.46
	sum	1.75 ± 0.22	1.69 ± 0.57	1.73 ± 0.37	0.57	0.50	0.94

Mean ± SEM(n) individual and sum of hydroxy-, dihydroxy- and epoxy-EPAs, -DHAs and -ARAs. P values are shown for genotype, parent PUFA and genotype*parent PUFA interaction using a univariate GLM. Age, gender, and BMI were used as covariates.

* *Statistically significant, P < 0.05*.

### Changes in Parent PUFAs and Their Derived Plasma Oxylipins Following Supplementation

**Figure 2:**
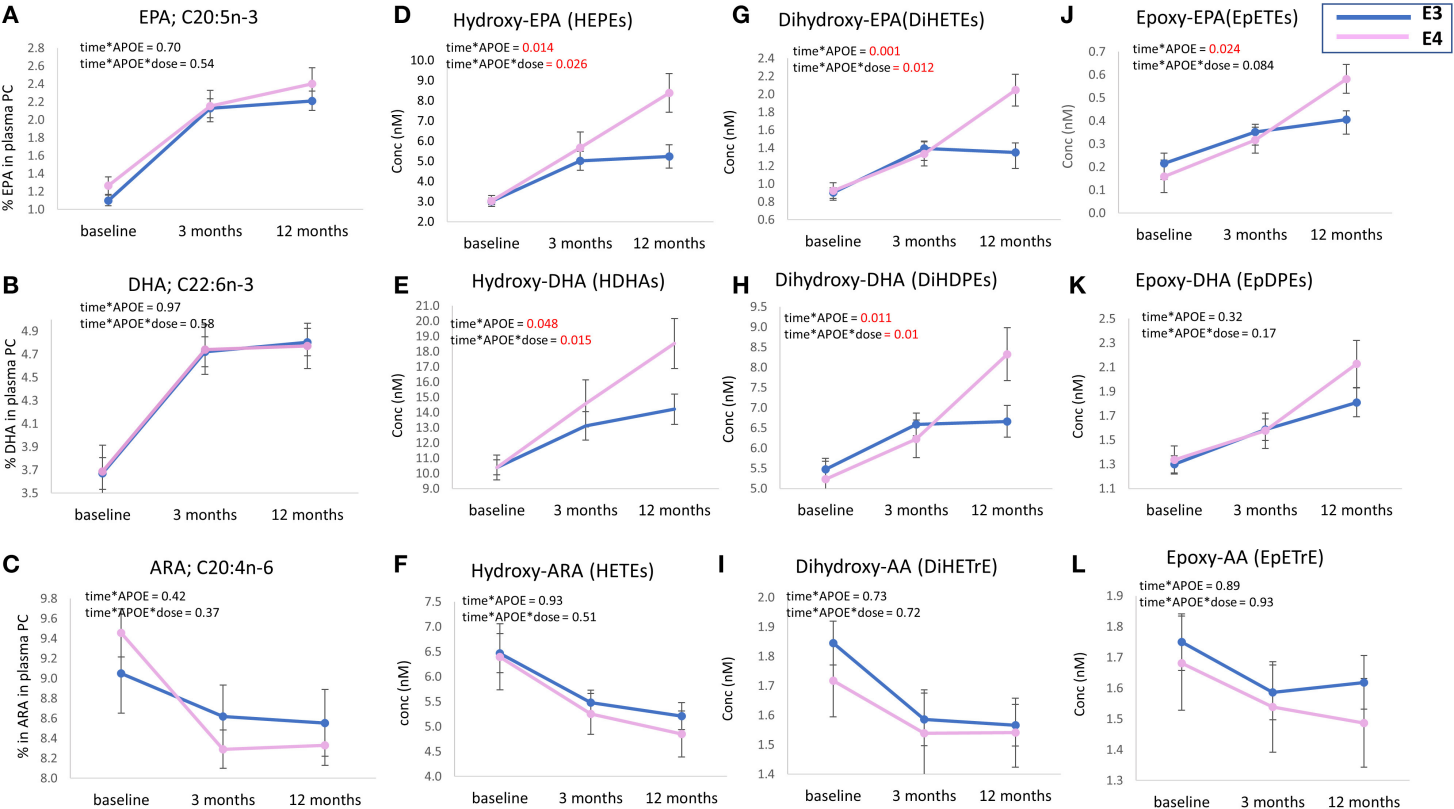
Plasma levels of parent PUFAs (EPA, DHA, and ARA) **(A–C)** and sum of EPA, DHA, and ARA-derived oxylipins; **(D–L)**, at baseline, 3 m and 12 m. Levels of EPA, DHA, and ARA (% of total fatty acids) and concentrations of plasma oxylipins (nM) are presented as mean ± SEM. Data were analyzed by repeated measures ANOVA. A model was built to identify the independent (main) effect of *APOE* and dose, and “APOE*dose” interaction effect. Age, sex, BMI, and baseline parent PUFA for oxylipins were used as covariates in the model. Within subject factor: time. The absolute concentrations of all HEPEs, HDHAs, DiHETEs, DiHDPEs, EpETEs, and EpDPEs covered by the analytical method were summed from the individual data, i.e., 9 × HEPEs, 10 × HDHAs, 4 × DiHETEs, 5 × DiHDPEs, 3 × EpETEs, and 4 × EpDPEs. APOE3 *n* = 66, APOE4 *n* = 26.

There was no difference in the level of plasma PC EPA, DHA, or ARA between *APOE3* and *APOE4* groups after 3 or 12 months of n-3 PUFA supplementation (Figures 2A–C). However, higher concentrations of EPA and DHA-derived oxylipins were observed in the *APOE4* group compared to the *APOE3* group. Differences were observed at 12 months (Figures 2D,E,G,H,J,K) for the sum of HEPEs, DiHETEs, EpETEs, HDHAs and DiHDPEs (*p* = 0.014, *p* = 0.001, *p* = 0.024, *p* = 0.048, and *p* = 0.011, respectively). There was no significant difference between *APOE3* and *APOE4* in the concentrations of epoxy-DHAs (Figure 2K) or epoxy-ARAs (Figures 2F,I,L). Analysis of n-3 PUFA^*^APOE (independent and interactive) was carried out on select LA- ALA- and DGLA- oxylipins. There were no significant effects evident (data not shown).

**Figure 3:**
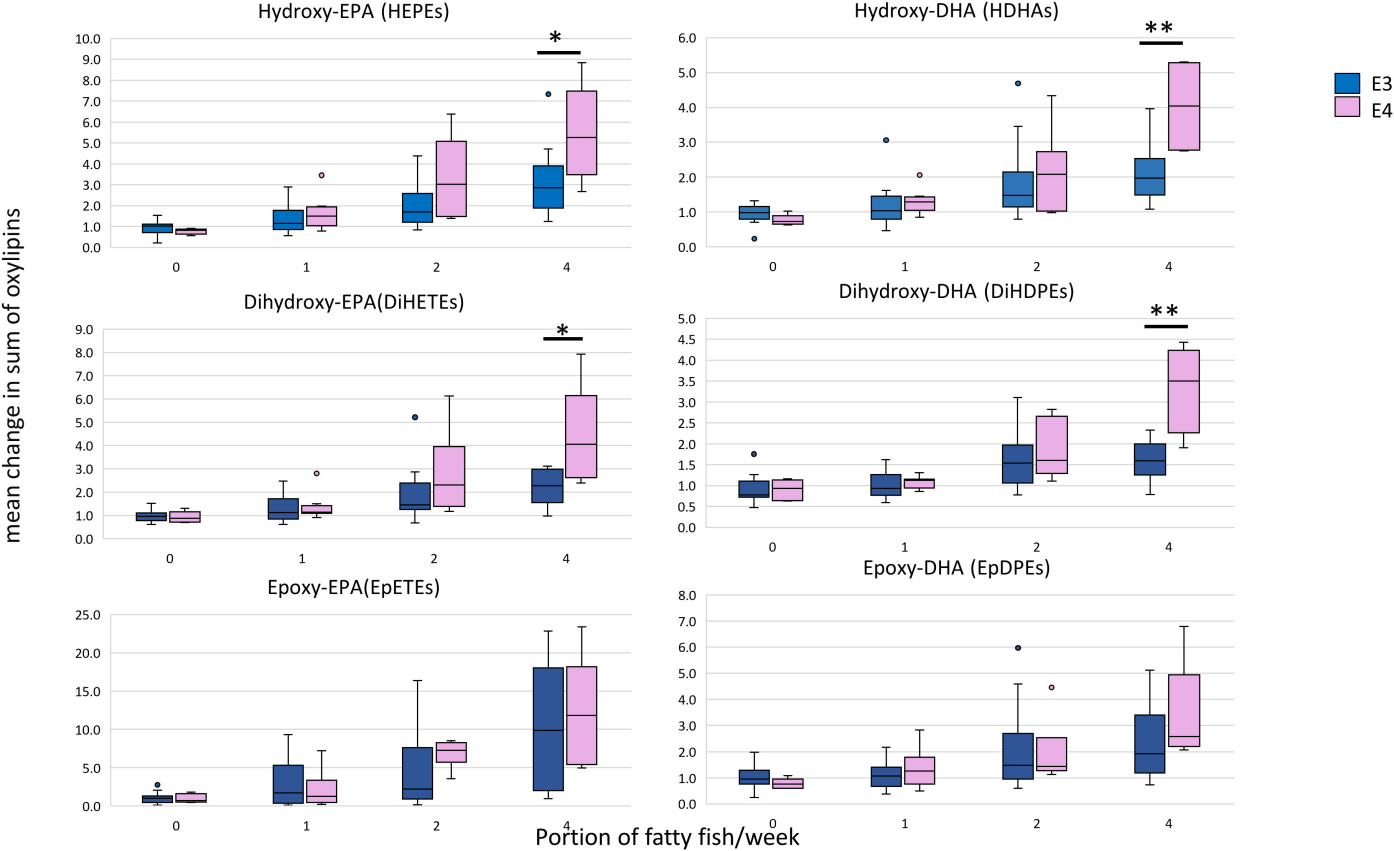
Box plots showing the mean relative change in oxylipin levels at 12 months with EPA+DHA intake, stratified according to *APOE* genotype. The concentrations of all HEPEs, HDHAs, EpETEs, and EpDPEs covered by the analytical method were summed from the individual data, i.e., 9 × HEPEs, 10 × HDHAs, 4 × DiHETEs, 5 × DiHDPEs, 3 × EpETEs, and 4 × EpDPEs. Mann-Witney test was used to compare APOE genotypes within each dose. *Statistically significant, *P* < 0.05; **Statistically significant, *P* < 0.01. APOE3 *n* = 66, APOE4 *n* = 26.

Considering the change in the sum of oxylipins derived from EPA and DHA at 12 months, a linear dose-response increase in oxylipins was observed as described previously (22). For the hydroxy- and dihydroxy-EPAs and -DHAs, but not for the epoxy-EPAs and -DHAs, the increase was significantly greater in the *APOE4* carriers who received the highest n-3 PUFA dose (equivalent to 4 portions of fatty fish/week) (Figure 3).

**Figure 4:**
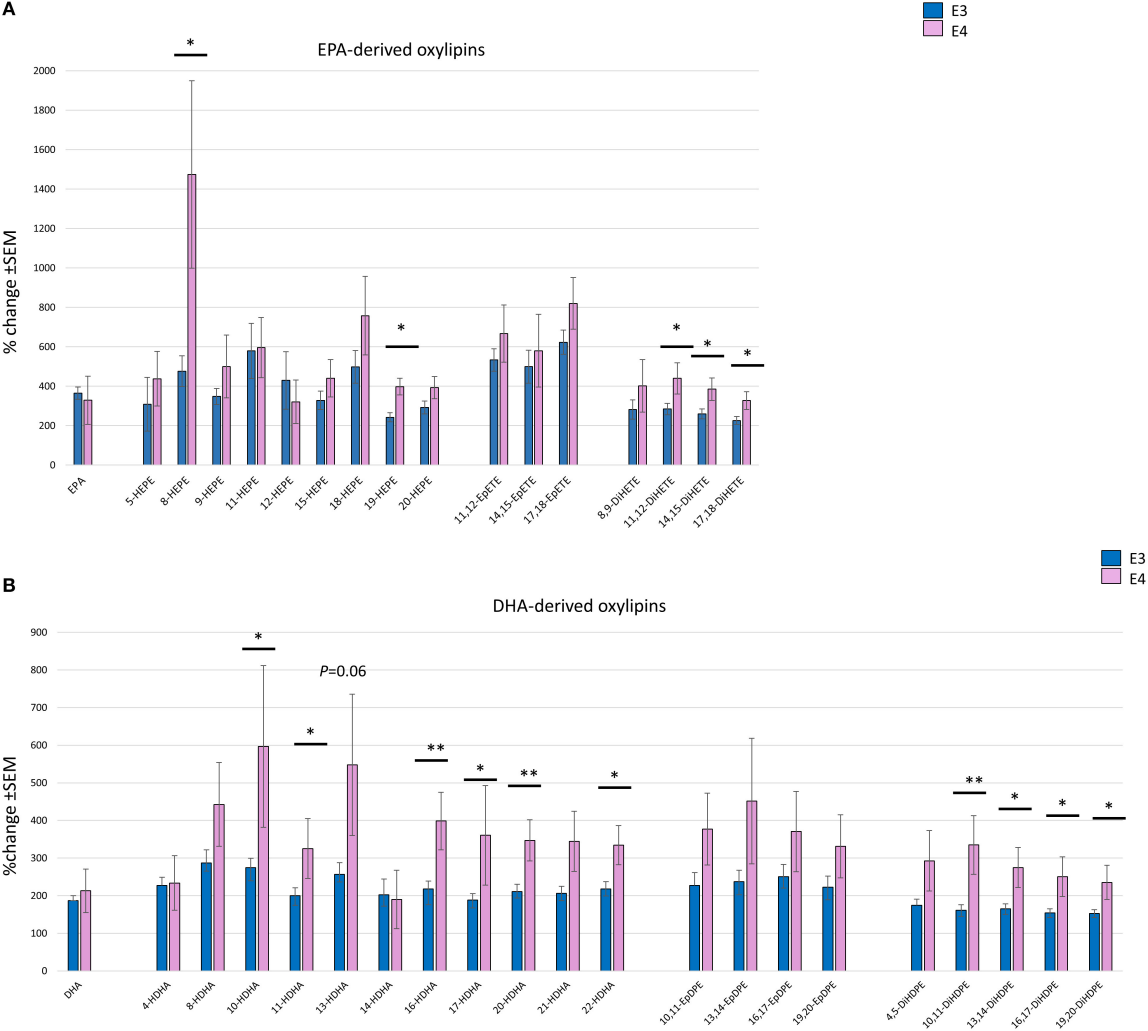
Bar charts showing the mean % change (with SEM) in individual EPA- **(A)** and DHA- **(B)** derived oxylipins, with their parent EPA and DHA following 12 months of supplementation with the equivalent of 4 portions of oily fish, comparing between *APOE3* (*n* = 17) and *APOE4* (*n* = 5/6). Independent sample *t*-test or Mann-Witney test was used to compare between *APOE* genotypes. *Statistically significant, *P* < 0.05, **Statistically significant, *P* < 0.01.

A focused analysis of the change in the parent PUFA and the corresponding oxylipins at the highest dose of n-3 PUFAs supplemented (equivalent to 4 portions of fatty fish/week) was done. Change in the parent EPA was higher (359% ± 32) than DHA (192% ± 14), with no difference observed between *APOE3* and *APOE4* groups (Figures 4A,B). The increase in EPA- and DHA-derived oxylipins was generally higher than their parent PUFA. A greater increase in almost all oxylipins was observed in the *APOE4* group, with the highest % change seen in the EPA-derived 8-HEPE (1,474% in E4 compared to 477% in E3) (*p* = 0.014) (Figure 4A). With regard to DHA-derived oxylipins, the highest % change was seen for 10-HDHA (597% in E4 compared to 274% in E3, *p* = 0.026) (Figure 4B). There was no significant difference in the epoxy-EPAs and -DHAs between *APOE3* and *APOE4* groups. After adjusting for age, sex, BMI and the basal level of parent n-3 PUFA, significant genotype^*^dose interactions were observed for two EPA-derived oxylipins: 19-HEPE and 20-HEPE (*p* = 0.027 and 0.011 (Table 4).

**Table 4:** Change from baseline in plasma oxylipins (nM) after 12 months of supplementation with n-3 PUFAs equivalent to 0, 1, 2, and 4 portions of fatty fish per week.

**Oxylipin**	**Portion of fish oil per week equivalency**												**P*_***APOE***_***	**P*_***dose***_***	P_APOE*dose_	**P_***baselineparent***_ _***PUFA***_**
	***APOE3***	***APOE4***	**Total**	***APOE3***	***APOE4***	**Total**	***APOE3***	***APOE4***	**Total**	***APOE3***	***APOE4***	**Total**				
	**(*n* = 14)**	**(*n* = 5)**	**(*n* = 19)**	**(*n* = 17)**	**(*n* = 10)**	**(*n* = 27)**	**(*n* = 17)**	**(*n* = 6)**	**(*n* = 23)**	**(*n* = 17)**	**(*n* = 5/6)**	**(*n* = 22/23)**				
**Hydroxy-EPA**																
5-HEPE	−0.04 ± 0.03	−0.08 ± 0.05	−0.05 ± 0.03	0.14 ± 0.07	0.12 ± 0.08	0.14 ± 0.05	0.25 ± 0.06	0.52 ± 0.19	0.32 ± 0.07	0.67 ± 0.08	0.57 ± 0.17	0.66 ± 0.07	0.463	<0.001	0.117	0.001
8-HEPE	−0.02 ± 0.02	−0.01 ± 0.01	−0.02 ± 0.01	0.09 ± 0.04	0.31 ± 0.20	0.17 ± 0.08	0.12 ± 0.04	0.28 ± 0.16	0.16 ± 0.05	0.27 ± 0.06	0.70 ± 0.08	0.33 ± 0.06	0.004	0.001	0.486	0.104
9-HEPE	0.00 ± 0.05	−0.07 ± 0.04	−0.02 ± 0.04	0.14 ± 0.06	0.27 ± 0.15	0.19 ± 0.06	0.23 ± 0.05	0.50 ± 0.18	0.30 ± 0.06	0.55 ± 0.07	0.92 ± 0.14	0.60 ± 0.07	0.006	<0.001	0.259	0.005
11-HEPE	−0.03 ± 0.02	−0.01 ± 0.01	−0.03 ± 0.01	0.11 ± 0.03	0.13 ± 0.07	0.12 ± 0.03	0.13 ± 0.03	0.29 ± 0.14	0.17 ± 0.04	0.30 ± 0.05	0.50 ± 0.13	0.33 ± 0.05	0.012	<0.001	0.207	0.005
12-HEPE	−0.02 ± 0.04	−0.06 ± 0.06	−0.03 ± 0.03	0.04 ± 0.09	0.31 ± 0.16	0.14 ± 0.08	0.38 ± 0.13	0.95 ± 0.42	0.53 ± 0.15	0.52 ± 0.16	0.27 ± 0.22	0.49 ± 0.14	0.256	0.002	0.196	0.114
15-HEPE	<0.13	<0.13	<0.13	<0.13	<0.13	<0.13	<0.13	<0.13	<0.13	0.17 ± 0.09	0.33 ± 0.33	0.19 ± 0.09	0.527	0.022	0.385	0.356
18-HEPE	−0.01 ± 0.02	0.01 ± 0.04	0 ± 0.01	0.11 ± 0.04	0.71 ± 0.42	0.33 ± 0.16	0.2 ± 0.04	0.37 ± 0.17	0.24 ± 0.05	0.58 ± 0.1	1.32 ± 0.56	0.69 ± 0.12	0.008	0.001	0.265	0.767
19-HEPE	−0.05 ± 0.12	−0.13 ± 0.07	−0.07 ± 0.09	0.21 ± 0.15	0.17 ± 0.09	0.20 ± 0.10	0.7 ± 0.22	1.68 ± 0.64	0.96 ± 0.24	1.36 ± 0.24	2.02 ± 0.2	1.45 ± 0.21	0.026	<0.001	0.027	0.024
20-HEPE	−0.05 ± 0.09	−0.29 ± 0.17	−0.11 ± 0.08	0.25 ± 0.11	0.11 ± 0.07	0.20 ± 0.07	0.42 ± 0.11	0.95 ± 0.38	0.56 ± 0.13	1.10 ± 0.13	1.33 ± 0.09	1.13 ± 0.12	0.198	<0.001	0.011	0.008
**Dihydroxy-EPA**																
8,9-DiHETE	−0.03 ± 0.01	−0.00 ± 0.01 0	−0.02 ± 0.01	0.02 ± 0.01	0.07 ± 0.03	0.04 ± 0.01	0.04 ± 0.02	0.10 ± 0.04	0.06 ± 0.02	0.13 ± 0.02	0.17 ± 0.03	0.14 ± 0.02	0.007	<0.001	0.633	0.003
11,12-DiHETE	−0.01 ± 0.01	0.00 ± 0.01	−0.01 ± 0.01	0.01 ± 0.01	0.07 ± 0.04	0.03 ± 0.02	0.04 ± 0.01	0.07 ± 0.02	0.05 ± 0.01	0.09 ± 0.01	0.18 ± 0.01	0.10 ± 0.01	0.002	<0.001	0.642	0.07
14,15-DiHETE	−0.01 ± 0.01	−0.01 ± 0.01	−0.01 ± 0.01	0.02 ± 0.01	0.08 ± 0.05	0.04 ± 0.02	0.07 ± 0.02	0.11 ± 0.03	0.08 ± 0.01	0.13 ± 0.02	0.26 ± 0.03	0.15 ± 0.02	0.001	<0.001	0.360	0.023
17,18-DiHETE	−0.04 ± 0.06	−0.01 ± 0.07	−0.03 ± 0.05	0.15 ± 0.09	0.39 ± 0.21	0.24 ± 0.10	0.46 ± 0.12	0.83 ± 0.25	0.55 ± 0.11	0.72 ± 0.1	1.45 ± 0.17	0.83 ± 0.10	0.001	<0.001	0.236	0.004
**Epoxy-EPA**																
11 (12)-EpETE	<0.05	<0.05	<0.05	0.03 ± 0.01	0.02 ± 0.01	0.030	0.06 ± 0.01	0.07 ± 0.02	0.06 ± 0.01	0.13 ± 0.02	0.17 ± 0.04	0.14 ± 0.02	0.605	<0.001	0.302	0.343
14 (15)-EpETE	0 ± 0.01	−0.01 ± 0.01	0 ± 0.01	0.02 ± 0.01	0.02 ± 0.02	0.02 ± 0.01	0.09 ± 0.02	0.11 ± 0.04	0.09 ± 0.02	0.17 ± 0.03	0.23 ± 0.04	0.18 ± 0.02	0.450	<0.001	0.818	0.419
17 (18)-EpETE	<0.1	<0.1	<0.1	<0.1	<0.1	<0.1	0.14 ± 0.03	0.21 ± 0.06	0.16 ± 0.03	0.26 ± 0.03	0.36 ± 0.07	0.28 ± 0.03	0.067	<0.001	0.294	0.213
**Hydroxy-DHA**																
4-HDHA	−0.08 ± 0.07	0.01 ± 0.14	−0.05 ± 0.06	0.06 ± 0.07	0.14 ± 0.09	0.09 ± 0.06	0.26 ± 0.08	0.36 ± 0.12	0.29 ± 0.07	0.48 ± 0.06	0.45 ± 0.17	0.47 ± 0.05	0.458	<0.001	0.994	0.688
7-HDHA	<0.1	<0.1	<0.1	0.13 ± 0.02	0.11 ± 0.02	0.12 ± 0.02	0.16 ± 0.02	0.23 ± 0.09	0.18 ± 0.03	0.25 ± 0.03	0.31 ± 0.06	0.26 ± 0.03	0.301	<0.001	0.54	0.605
8-HDHA	−0.08 ± 0.09	−0.15 ± 0.12	−0.1 ± 0.07	0.21 ± 0.09	0.28 ± 0.11	0.24 ± 0.07	0.37 ± 0.09	0.68 ± 0.31	0.45 ± 0.10	0.82 ± 0.12	1.34 ± 0.21	0.90 ± 0.11	0.062	<0.001	0.458	0.514
10-HDHA	−0.02 ± 0.01	−0.01 ± 0.03	−0.02 ± 0.01	0.03 ± 0.02	0.11 ± 0.04	0.06 ± 0.02	0.1 ± 0.02	0.19 ± 0.10	0.12 ± 0.03	0.19 ± 0.02	0.35 ± 0.02	0.21 ± 0.02	0.008	<0.001	0.577	1.000
11-HDHA	−0.03 ± 0.02	−0.02 ± 0.05	−0.03 ± 0.02	0.01 ± 0.03	0.14 ± 0.04	0.06 ± 0.03	0.15 ± 0.04	0.27 ± 0.10	0.18 ± 0.04	0.21 ± 0.04	0.34 ± 0.01	0.23 ± 0.03	0.016	<0.001	0.658	0.717
13-HDHA	−0.01 ± 0.01	0.01 ± 0.03	−0.01 ± 0.01	0.04 ± 0.02	0.05 ± 0.02	0.05 ± 0.02	0.08 ± 0.02	0.14 ± 0.09	0.10 ± 0.03	0.15 ± 0.02	0.31 ± 0.09	0.17 ± 0.03	0.024	<0.001	0.352	0.369
14-HDHA	0.09 ± 0.16	−0.42 ± 0.43	−0.04 ± 0.16	−0.38 ± 0.32	1.45 ± 0.91	0.3 ± 0.42	1.04 ± 0.45	2.7 ± 1.35	1.47 ± 0.49	0.61 ± 0.34	0.32 ± 0.82	0.57 ± 0.31	0.21	0.015	0.096	0.106
16-HDHA	−0.02 ± 0.01	−0.01 ± 0.04	−0.02 ± 0.01	0.05 ± 0.02	0.14 ± 0.06	0.08 ± 0.03	0.10 ± 0.03	0.17 ± 0.09	0.12 ± 0.03	0.18 ± 0.03	0.41 ± 0.04	0.22 ± 0.03	0.003	<0.001	0.312	0.412
17-HDHA	−0.11 ± 0.07	−0.06 ± 0.17	−0.1 ± 0.06	0.06 ± 0.15	0.03 ± 0.18	0.05 ± 0.11	0.28 ± 0.11	0.67 ± 0.22	0.38 ± 0.10	0.63 ± 0.11	1.36 ± 0.08	0.73 ± 0.11	0.033	<0.001	0.155	0.455
20-HDHA	−0.06 ± 0.04	0.02 ± 0.13	−0.04 ± 0.04	0.09 ± 0.06	0.35 ± 0.17	0.18 ± 0.08	0.24 ± 0.05	0.38 ± 0.22	0.28 ± 0.07	0.43 ± 0.06	0.93 ± 0.10	0.50 ± 0.07	0.004	<0.001	0.429	0.666
21-HDHA	−0.10 ± 0.48	−1.08 ± 0.37	−0.36 ± 0.38	0.18 ± 0.33	0.26 ± 0.34	0.21 ± 0.24	1.62 ± 0.38	2.40 ± 1.13	1.82 ± 0.40	3.21 ± 0.47	4.68 ± 0.63	3.42 ± 0.42	0.357	<0.001	0.434	0.093
22-HDHA	−0.10 ± 0.41	0.03 ± 1.14	−0.07 ± 0.41	0.08 ± 0.35	0.12 ± 0.41	0.1 ± 0.26	1.29 ± 0.32	1.99 ± 0.97	1.47 ± 0.34	3.08 ± 0.48	4.08 ± 0.47	3.23 ± 0.42	0.209	<0.001	0.786	0.225
**Dihydroxy-DHA**																
4,5-DiHDPE	−0.23 ± 0.15	−0.19 ± 0.22	−0.22 ± 0.12	0.05 ± 0.16	0.10 ± 0.1	0.06 ± 0.11	0.92 ± 0.34	1.15 ± 0.27	0.98 ± 0.26	0.80 ± 0.19	1.81 ± 0.57	0.95 ± 0.19	0.144	<0.001	0.523	0.316
10,11-DiHDPE	−0.04 ± 0.02	−0.03 ± 0.04	−0.04 ± 0.02	0.00 ± 0.03	0.03 ± 0.02	0.01 ± 0.02	0.09 ± 0.03	0.18 ± 0.10	0.11 ± 0.03	0.12 ± 0.03	0.33 ± 0.07	0.15 ± 0.03	0.005	<0.001	0.228	0.089
13,14-DiHDPE	−0.04 ± 0.02	−0.04 ± 0.03	−0.04 ± 0.02	0.01 ± 0.02	0.16 ± 0.12	0.06 ± 0.05	0.11 ± 0.03	0.14 ± 0.05	0.12 ± 0.03	0.14 ± 0.03	0.41 ± 0.05	0.18 ± 0.03	0.004	<0.001	0.182	0.145
16,17-DiHDPE	−0.02 ± 0.02	−0.03 ± 0.03	−0.02 ± 0.02	−0.01 ± 0.02	0.16 ± 0.11	0.05 ± 0.04	0.16 ± 0.04	0.16 ± 0.07	0.16 ± 0.03	0.15 ± 0.03	0.42 ± 0.07	0.19 ± 0.04	0.008	<0.001	0.100	0.095
19,20-DiHDPE	−0.44 ± 0.29	−0.25 ± 0.32	−0.39 ± 0.22	0.02 ± 0.21	1.33 ± 1.10	0.51 ± 0.43	1.45 ± 0.37	1.72 ± 0.68	1.52 ± 0.32	1.39 ± 0.28	3.85 ± 0.77	1.74 ± 0.32	0.006	<0.001	0.349	0.054
**Epoxy-DHA**																
10 (11)-EpDPE	0.00 ± 0.04	−0.05 ± 0.03	−0.01 ± 0.03	0.00 ± 0.04	0.03 ± 0.05	0.02 ± 0.03	0.16 ± 0.06	0.24 ± 0.10	0.18 ± 0.05	0.31 ± 0.09	0.43 ± 0.09	0.32 ± 0.07	0.496	<0.001	0.931	0.37
13 (14)-EpDPE	−0.01 ± 0.03	−0.06 ± 0.02	−0.03 ± 0.02	0.00 ± 0.03	0.06 ± 0.05	0.03 ± 0.03	0.15 ± 0.05	0.15 ± 0.10	0.15 ± 0.04	0.27 ± 0.07	0.46 ± 0.12	0.29 ± 0.06	0.394	<0.001	0.452	0.387
16 (17)-EpDPE	−0.02 ± 0.03	−0.02 ± 0.05	−0.02 ± 0.03	0.00 ± 0.04	0.04 ± 0.05	0.02 ± 0.03	0.15 ± 0.05	0.16 ± 0.08	0.15 ± 0.04	0.30 ± 0.07	0.41 ± 0.10	0.32 ± 0.06	0.566	<0.001	0.886	0.681
19 (20)-EpDPE	−0.01 ± 0.05	−0.10 ± 0.04	−0.03 ± 0.04	0.02 ± 0.06	0.07 ± 0.06	0.04 ± 0.04	0.25 ± 0.08	0.37 ± 0.19	0.29 ± 0.08	0.47 ± 0.11	0.69 ± 0.10	0.50 ± 0.10	0.405	<0.001	0.862	0.301
**Hydroxy-ARA**																
5-HETE	−0.28 ± 0.23	−0.26 ± 0.10	−0.27 ± 0.17	−0.14 ± 0.08	−0.21 ± 0.16	−0.16 ± 0.08	−0.06 ± 0.24	−0.29 ± 0.07	−0.12 ± 0.18	−0.24 ± 0.10	−0.32 ± 0.36	−0.25 ± 0.10	0.187	0.928	0.924	0.349
8-HETE	−0.02 ± 0.02	−0.02 ± 0.02	−0.02 ± 0.01	−0.02 ± 0.02	0.01 ± 0.03	−0.01 ± 0.02	−0.03 ± 0.02	−0.01 ± 0.03	−0.03 ± 0.02	−0.04 ± 0.01	−0.02 ± 0.01	−0.04 ± 0.01	0.600	0.684	0.971	0.674
11-HETE	−0.03 ± 0.01	−0.07 ± 0.02	−0.04 ± 0.01	0.00 ± 0.02	0.00 ± 0.05	0.00 ± 0.02	−0.04 ± 0.03	−0.02 ± 0.02	−0.04 ± 0.02	−0.06 ± 0.01	−0.05 ± 0.03	−0.06 ± 0.01	0.545	0.181	0.784	0.357
12-HETE	0.09 ± 0.39	−0.65 ± 0.54	−0.11 ± 0.33	0.07 ± 0.45	0.21 ± 0.66	0.12 ± 0.37	−0.26 ± 0.49	−0.54 ± 1.26	−0.33 ± 0.47	−0.61 ± 0.27	−0.05 ± 0.06	−0.53 ± 0.23	0.332	0.790	0.997	0.239
15-HETE	−0.09 ± 0.06	−0.23 ± 0.09	−0.13 ± 0.05	−0.10 ± 0.07	−0.13 ± 0.06	−0.11 ± 0.05	−0.17 ± 0.06	−0.14 ± 0.05	−0.16 ± 0.05	−0.28 ± 0.05	−0.12 ± 0.11	−0.25 ± 0.05	0.634	0.546	0.391	0.131
20-HETE	0.04 ± 0.09	−0.08 ± 0.14	0.01 ± 0.08	−0.10 ± 0.08	−0.25 ± 0.08	−0.16 ± 0.06	−0.18 ± 0.05	0.10 ± 0.18	−0.10 ± 0.06	−0.22 ± 0.08	0.15 ± 0.22	−0.17 ± 0.08	0.827	0.517	0.051	0.791
**Dihydroxy-ARA**																
5,6-DiHETrE	−0.18 ± 0.15	−0.02 ± 0.01	−0.14 ± 0.11	−0.02 ± 0.03	−0.01 ± 0.03	−0.02 ± 0.02	0.02 ± 0.15	0 ± 0.04	0.02 ± 0.11	−0.08 ± 0.04	−0.08 ± 0.1	−0.08 ± 0.04	0.544	0.717	0.991	0.765
8,9-DiHETrE	−0.03 ± 0.03	0 ± 0.02	−0.02 ± 0.02	−0.03 ± 0.01	−0.04 ± 0.02	−0.03 ± 0.01	−0.02 ± 0.03	0.01 ± 0.04	−0.02 ± 0.03	−0.08 ± 0.02	−0.02 ± 0.03	−0.07 ± 0.02	0.613	0.224	0.735	0.657
11,12-DiHETrE	−0.04 ± 0.03	−0.03 ± 0.04	−0.04 ± 0.03	−0.06 ± 0.02	−0.16 ± 0.03	−0.1 ± 0.02	−0.08 ± 0.03	−0.04 ± 0.04	−0.07 ± 0.02	−0.19 ± 0.03	−0.01 ± 0.08	−0.17 ± 0.03	0.369	0.164	0.02	0.459
14,15-DiHETrE	−0.02 ± 0.04	−0.03 ± 0.06	−0.02 ± 0.03	−0.07 ± 0.02	−0.14 ± 0.02	−0.09 ± 0.02	−0.06 ± 0.03	−0.05 ± 0.04	−0.06 ± 0.02	−0.18 ± 0.04	−0.02 ± 0.09	−0.16 ± 0.04	0.648	0.132	0.193	0.38
**Epoxy-ARA**																
5 (6)-EpETrE	0.08 ± 0.12	−0.11 ± 0.16	0.03 ± 0.1	−0.13 ± 0.12	−0.06 ± 0.15	−0.1 ± 0.1	−0.09 ± 0.12	0.05 ± 0.14	−0.05 ± 0.1	−0.13 ± 0.13	−0.06 ± 0.25	−0.12 ± 0.12	0.673	0.636	0.903	0.998
8 (9)-EpETrE	0 ± 0.02	0 ± 0.04	0 ± 0.02	−0.02 ± 0.02	0 ± 0.03	−0.01 ± 0.02	−0.01 ± 0.03	0.01 ± 0.04	0 ± 0.02	0 ± 0.02	0 ± 0.03	0 ± 0.02	0.736	0.928	0.916	0.635
11 (12)-EpETrE	−0.01 ± 0.02	−0.01 ± 0.02	−0.01 ± 0.01	−0.03 ± 0.02	−0.02 ± 0.03	−0.03 ± 0.02	0 ± 0.02	0 ± 0.05	0 ± 0.02	−0.01 ± 0.02	0.03 ± 0.01	0 ± 0.02	0.861	0.538	0.878	0.766
14 (15)-EpETrE	−0.01 ± 0.05	−0.01 ± 0.07	−0.01 ± 0.04	−0.11 ± 0.04	−0.07 ± 0.07	−0.09 ± 0.03	−0.03 ± 0.05	−0.02 ± 0.1	−0.03 ± 0.04	−0.03 ± 0.04	0.09 ± 0.05	−0.02 ± 0.04	0.393	0.223	0.639	0.582

Mean ± SEM(n) absolute change in individual hydroxy-, dihydroxy- and epoxy-EPAs, -DHAs and -ARAs. P values are shown for genotype, dose PUFA and genotype^*^dose interaction using univariate GLM. P value for baseline parent PUFA was calculated in the same univariate GLM as a covariate. Other covariates used are age, gender and BMI.

### Effect of *APOE* Genotype Is More Evident for the Dihydroxy-EPAs and -DHAs Compared to the Epoxy-EPAs and -DHAs

A strong independent effect of *APOE* genotype on all dihydroxy-EPAs and -DHAs (except 4,5 DiHDPE) was observed (Table 4). Despite the higher levels of epoxy-EPAs and -DHAs in the *APOE4* group, the differences were not statistically significant (Table 4 and Figures 4A,B).

### Influence of the Basal Parent Plasma PUFA on the Change in Plasma Oxylipins Is More Pronounced for EPA-Derived Oxylipins

**Figure 5:**
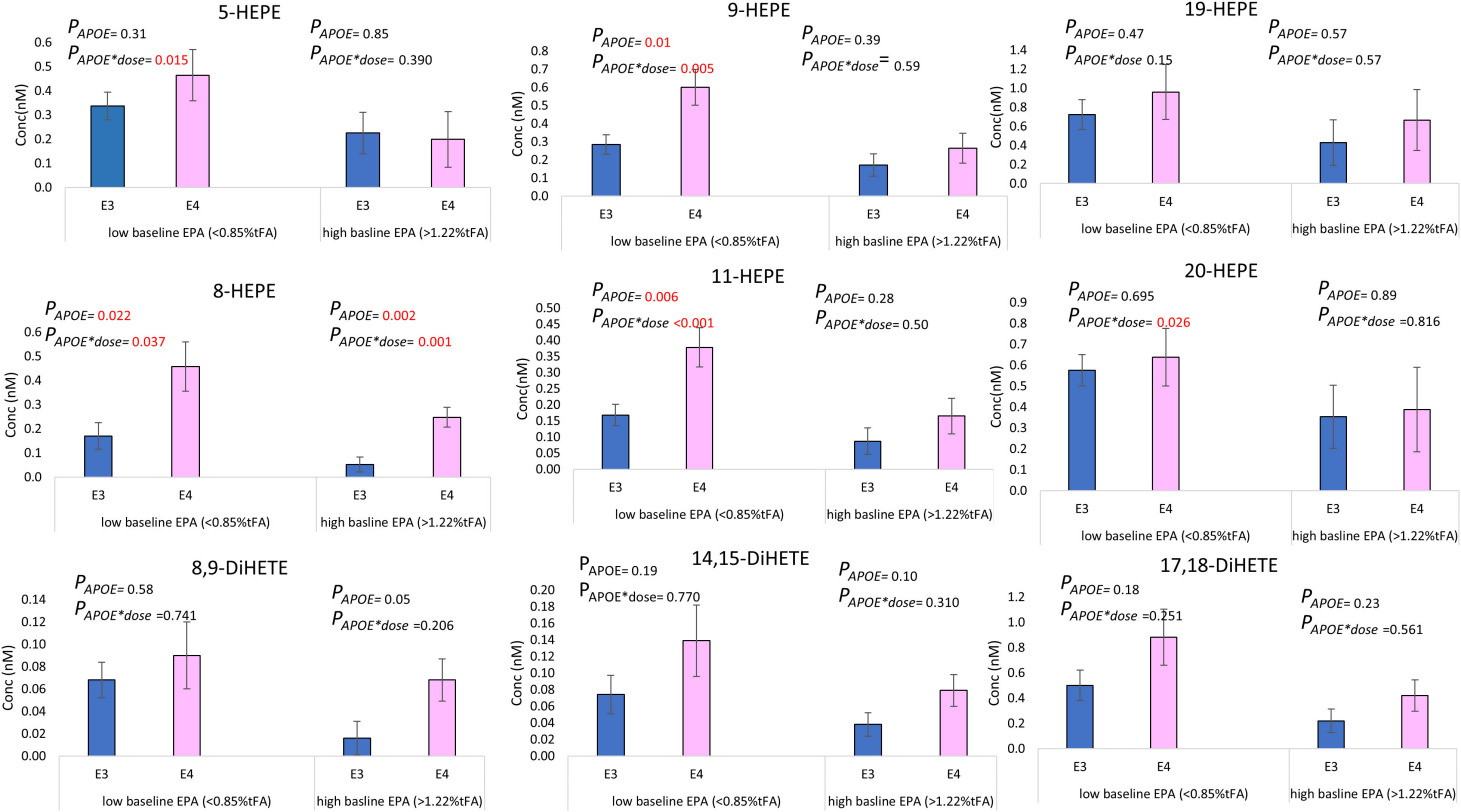
Mean absolute change (±SEM) in select EPA-derived oxylipins (nM) at 12 months of supplementation, according to *APOE* genotype and the level of baseline EPA. Low basal EPA (*APOE3 n* = 22, *APOE4 n* = 9), high basal EPA (*APOE3 n* = 18, *APOE4 n* = 10).

The basal level of plasma PC EPA had a significant effect on the change in concentration of select hydroxy- and dihydroxy-EPAs (Table 4) but not epoxy-EPAs. When dividing baseline plasma PC EPA levels into tertiles and different, a higher change in 5-HEPE, 9-HEPE, 11-HEPE, and 20-HEPE was observed at a low basal EPA level (EPA <0.85% of total fatty acids) compared to high basal EPA level (EPA > 1.22% of total fatty acids) in *APOE4* carriers (Figure 5). On the other hand, the basal level of plasma PC DHA had no influence on the change in DHA-derived oxylipins (Table 4).

## Discussion

PUFAs mediate inflammatory status partly through the balance between n-6 PUFA-derived and n-3 PUFA-derived oxylipins (9). Recent studies show a linear response of EPA- and DHA-derived oxylipins to fish oil supplementation (24, 25). However, a strong inter-individual variation is observed. *APOE* genotype is known to modulate systemic inflammation and neuroinflammation, and the response to fish oil interventions, in healthy subjects (31) and in patients with cardiovascular (32) and cognitive disorders (33). To our knowledge, no previous studies have explored the effect of *APOE* genotype on oxylipins, either cross-sectionally or in response to n-3 PUFA supplementation. In the current study, we show that *APOE* genotype affects the plasma oxylipin concentrations and their response to EPA+DHA intervention in healthy participants. We observe higher levels of hydroxy- and dihydroxy-EPA- and DHA-derived oxylipins in *APOE4* carriers compared to the wild type *APOE3/E3* genotype. This difference becomes more evident with higher doses of n-3 PUFAs supplemented for longer periods (12 months). The greatest increase was in 8-HEPE, an oxylipin formed by autoxidation. It has recently been shown that 8-HEPE, together with other HEPEs, has a high ligand activity for PPARs (40, 41). A significant effect of *APOE* on dihydroxy- but not the epoxy-EPAs and -DHAs suggests a more active sEH enzyme in *APOE4* carriers. Analyzing samples from a well-designed intervention trial, with different doses of n-3 PUFAs and for a duration up to 12 months, highlights the importance of n-3 PUFA dose and duration of intake in modulating select plasma oxylipin levels such as 8-HEPE and 17-HDHA. Higher levels of these oxylipins in *APOE4* carriers, may help mitigate a more disrupted metabolic and pro-inflammatory profile relative to the common *APOE3/3* in multiple disease pathologies (42, 43).

Prior to intervention, there was no difference in the levels of ARA, EPA and DHA between *APOE3* and *APOE4* carriers (Table 3). This is consistent with the Multi-Ethnic Study of Atherosclerosis (MESA), where, although there was no difference in plasma phospholipid EPA and DHA concentrations between *APOE3* and *APOE4* groups an APOE^*^n-3 PUFA interaction was evident with high density lipoprotein cholesterol and particle size (44). Similarly, in the Alzheimer's Disease Cooperative Study there were no differences between *APOE* genotypes for EPA and DHA in plasma phospholipids at baseline (45). In contrast, Plourde et al. showed that EPA and DHA were higher in *APOE4* carriers, in plasma triglycerides while there was no differences in n-3 PUFAs between genotypes in the non-esterified fatty acid fraction, with plasma phospholipid fraction composition data not included (46).

Similar to baseline, there was no difference in the levels of EPA, DHA or ARA between *APOE3* and *APOE4* groups after 12 months of n-3 PUFA supplementation (Figures 2A–C), which is consistent with previous n-3 PUFA intervention studies carried out in healthy subjects (47, 48) and after 18 months of DHA supplementation in patients with Alzheimer's disease (49). However, in some studies, the ratios of DHA/AA and EPA/AA were lower in the *APOE4* group following n-3 PUFA supplementation (45). This was not found in the current analysis (data not shown).

At baseline, there was no difference in plasma oxylipin levels between *APOE3* and *APOE4* except for 11-HDHA and 20-HETE, which were both lower in the *APOE4* group (Table 3). In mouse models, there was no significant difference in the level of 20-HETE between wild type and *APOE* knockout mice. However, after being fed with a high fat diet, 20-HETE was higher in the renal tissue of the *APOE* knockout mice, with no difference observed in the hepatic tissue (50). Similarly, in a mouse model of abdominal aortic aneurysm, levels of several HETEs (5-, 8-, 12-, 15-HETE) were similar in the blood of wildtype and *APOE* knockout mice, and were higher in *APOE* knockout mice after pro-coagulant administration (51). In aged mice, brain cortical levels of EPA-derived 18-HEPE and DHA-derived 10,17-diHDHA, together with the specialized pro-resolving mediator resolvin D1, were lower in *APOE4* mice compared to *APOE3* (52).

The changes in plasma EPA- and DHA-oxylipins were consistently higher in *APOE4* compared to *APOE3/E3* (Figures 4A,B), with the highest change observed for 8-HEPE (1,474% in *APOE4* vs. 477% in *APOE3, p* = 0.014) (Figure 4A). Most plasma oxylipins are bound to lipoproteins (53), and in particular LDL (54). *APOE4* has a higher affinity for the LDL-receptor leading to increased catabolism of VLDL and a subsequent increase in LDL-cholesterol (55, 56). In addition, n-3 PUFA supplementation was found to increase LDL-cholesterol concentrations (57), which becomes more pronounced in *APOE4* carriers, and more evident in chronic inflammatory conditions (32). Interestingly, after 1 year of 0.840 g/day EPA+DHA and vitamin D supplementation in a subset of the VITAL study, an association between some oxylipins and increased LDL was found (58). Consequently, although LDL data are not available in the current RCT, it is speculated that, with higher doses of EPA and DHA, the differential increase of oxylipins in *APOE4* carriers could relate to possibly higher LDL levels in those individuals. However, given the healthy status of the participants in this study, future studies focusing on patients with chronic inflammatory conditions are needed for confirmation.

Recent studies show that HEPEs have higher ligand activity for PPARs than their parent EPA (40, 41). 8-HEPE activated the transcription of PPARs leading to increased adipogenesis and cellular glucose uptake in fibroblasts and muscle cell-lines (41), and improved dyslipidemia in a PPARα-dependent manner (17). Several studies showed that glucose and lipid metabolism and fatty acid oxidation are disturbed in *APOE4* carriers (59–62). Interestingly, PPARγ signaling was also found to be disturbed in *APOE4* carriers (60, 63, 64). Taken together, we suggest that the post-n-3 PUFA intervention differential increase in levels of HEPEs observed here, especially in 8-HEPE, promotes PPARγ activation, and consequently could contribute to the partial mitigation of the disturbed metabolic processes evident in *APOE4* carriers.

*APOE4* individuals have a higher inflammatory status and more oxidative stress (65, 66) compared to *APOE3* carriers. LPS-stimulated macrophages from human and mice showed increased TNF-α and IL-6, and the activation of the inflammatory NFκB pathway in *APOE4* compared to *APOE3* carriers (65). We have previously shown higher levels of h-CRP, P-selectin and E-selectin in normal-weight healthy *APOE4* individuals in comparison to *APOE3/E3* (67). Similarly, in the mouse brain, an *APOE4* genotype was associated with increased microglial activation, IL-1β and lipid peroxidation (65, 66).

A possible mechanism for the increase in HEPEs and HDHAs in *APOE4* carriers could be the increased activity of 5- and 12/15-lipoxygenases and increased auto-oxidation in response to the higher inflammatory status in *APOE4* carriers. Increased activity of lipoxygenase enzymes was found in the macrophages and atherosclerotic plaques of *APOE* KO mice used as models of chronic inflammation (68, 69). Moreover, the absence of 12/15-lipoxygenase reduced oxidative stress in the brains of *APOE* KO mice (70). N-3 PUFA were shown to increase lipoxins and resolvins in atherosclerosis (71) and Alzheimer's disease (72), thus suggesting EPA/DHA-induced activation of 5- and 12/15-lipoxygenases. Similarly, products of n-3 PUFA autooxidation increase with n-3 PUFA supplementation, with studies showing their beneficial effects on cardiovascular diseases (8). Interestingly, in this study the 15-lipoxygenase derived oxylipin 17-HDHA was significantly higher in *APOE4* (*P*_APOE_ = 0.03) (Table 4 and Figure 4B). 17-HDHA is the precursor of D-resolvins and protectins which possess strong pro-resolving and anti-inflammatory properties and are dysregulated in several chronic inflammatory and neurodegenerative conditions (17, 52). Taken together, we suggest that the inflammatory environment in *APOE4* carriers could increase the activity of lipoxygenases and autooxidation of PUFA which in turn leads to an increased production of EPA-and DHA-oxylipins when fish oil is supplemented.

Consistent with previous observations of greater increases in oxylipins in those with lower parent PUFA at baseline (25, 73), we observed that the change in the EPA- oxylipins (5-, 9-, 11-, 19, 20-HEPEs-, and all DiHETEs), though not the DHA- oxylipins, was associated with baseline EPA levels in plasma PC. In an earlier study, we found that the increase in 5-HEPE and 17,18-DiHETE after 12 weeks of n-3 PUFA supplementation was significantly associated with baseline EPA status (*p* < 0.01 and *p* < 0.05, respectively), with higher levels observed in subjects with low baseline EPA levels (23). The current study shows that higher levels of 5-HEPE, 9-HEPE, 11-HEPE, and 20-HEPE are present in *APOE4* individuals with low basal EPA status, compared to *APOE3* (Figure 5).

APOE^*^time interactions were only evident for hydroxy- and dihydroxy-EPAs and -DHAs, with no significant increase in the epoxy-EPAs and DHAs (Figure 2 and Table 4). This is despite the increase of hydroxy-, dihydroxy- and epoxy-EPAs and -DHAs which was generally observed with n-3 PUFA intervention (Figure 2), Dihydroxy-oxylipins are the metabolic products of epoxy-oxylipins by the action of sEH, with the ratio of DiHETE/EpETE being indicative of sEH activity (13, 74).

It is important to note here the effect of n-3 PUFA dose and duration of intake on the changes in plasma oxylipins in *APOE4* carriers. The difference in n-3 PUFA-derived oxylipins was observed with an n-3 PUFA dose equivalent to two portions of oily fish intake per week and became more evident with the dose equivalent to four portions per week, at 12 months of n-3 PUFA supplementation. Indeed, it has been demonstrated that a threshold intake of n-3 PUFAs may be required before a favorable effect is observed, whether in the form of increased specialized pro-resolving mediators production (75) or in reducing neuroinflammation (76).

The main strengths of this study were the large number of oxylipins quantified, the dose response nature of the analysis (three physiologically relevant doses included), and the long intervention period of 12 months, which included interim assessment at 3 months.

The present study has some limitations. Due to the exploratory nature of the analysis, participants were genotyped retrospectively and an unequal sample size between *APOE3* (*n* = 66) and *APOE4* (*n* = 26) inevitable. When further subgrouping the participants according to n-3 PUFA dose, the numbers in the genotype groups were lower, reaching 5–6 in the four portions fatty fish/week group. However, an independent association of *APOE* with plasma oxylipins was still observed, regardless of the n-3 PUFA dose given. Another limitation is that correction for multiple testing was not applied, which may lead to overestimation of the findings. Due to the exploratory nature of this study, validation in a study with a larger number of participants is necessary.

In conclusion, this study shows for the first time the impact of *APOE* genotype on plasma oxylipin concentrations and their response to EPA+DHA intervention. Higher levels of EPA- and DHA- oxylipins in *APOE4* carriers compared to the wild type *APOE3/E3* genotype become more evident with higher doses of n-3 PUFAs supplemented for longer periods (12 months). The greatest increase was in autoxidatively formed 8-HEPE which is a PPAR activator, with PPAR activation shown previously to be inhibited in *APOE4* carriers (64). This study indicates that *APOE* genotype mediating oxylipin production, and may be an important contributor to the inter-individual variability and dose-response relationship between fish oil supplementation and health outcomes. Future studies should focus on the HEPEs-PPARs-glucose and lipid metabolism axis according to *APOE* genotype status, given their greater increase in response to PUFA supplementation in *APOE4* carriers and their emerging importance in regulating cellular metabolism.

## Data Availability Statement

The original contributions presented in the study are included in the article, further inquiries can be directed to the corresponding authors.

## Ethics Statement

The studies involving human participants were reviewed and approved by Suffolk Local Research Ethics Committee. The patients/participants provided their written informed consent to participate in this study.

## Author Contributions

PC and AW designed and carried out the original intervention study and conducted fatty acid analysis. AO and NS conducted the oxylipin measurements. RS and AM designed the current study, analyzed the data and performed statistical analysis, drafted the manuscript, and have primary responsibility for its final content. All authors read and approved the final manuscript.

## Funding

This clinical trial was funded by the UK Foods Standards Agency (grants N05065 and N05066) and the UK Medical Research Council (grants U105960389 and U1052.00.014). Oxylipin measurements were supported by the German Research Foundation (grant Schebb 1801). *APOE* genotyping and the current data analysis were funded by EU-JPI/BBSRC (BB/P028233/1). We acknowledge Susan Jebb, Celia Walker and Lucy Browning for their contribution to the clinical trial.

## Conflict of Interest

The authors declare that the research was conducted in the absence of any commercial or financial relationships that could be construed as a potential conflict of interest.

## Publisher's Note

All claims expressed in this article are solely those of the authors and do not necessarily represent those of their affiliated organizations, or those of the publisher, the editors and the reviewers. Any product that may be evaluated in this article, or claim that may be made by its manufacturer, is not guaranteed or endorsed by the publisher.
